# MR myocardial perfusion analysis of first-pass enhancement kinetics with a lagrangian approach

**DOI:** 10.1186/1532-429X-16-S1-P203

**Published:** 2014-01-16

**Authors:** Sohae Chung, Binita Shah, Sohah Iqbal, James Slater, Leon Axel

**Affiliations:** 1 Department of Radiology, NYU Langone Medical Center, New York, New York, USA; 2 Department of Medicine, Division of Cardiology, New York University School of Medicine, New York, New York, USA

## Background

**Figure 1:**
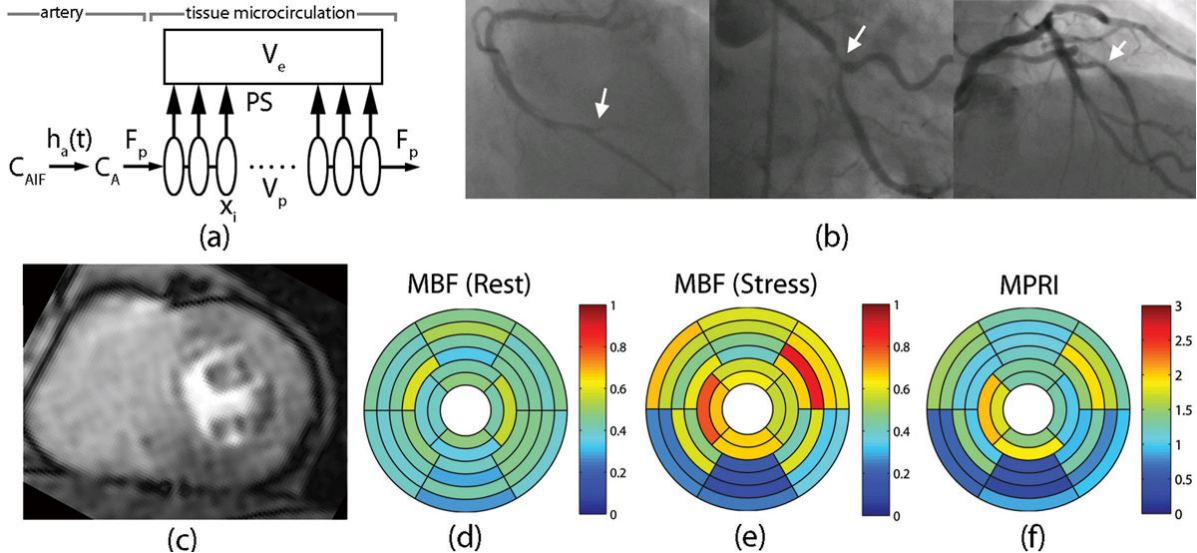
**(a) Schematics of the TH model with a Lagrangian approach**. (b) Selective coronary angiography in a representative patient with triple-vessel disease (from left to right, total occlusion of right AV groove, severe circumflex obtuse marginal bifurcation lesion, severe mid left anterior descending artery lesion). (c) Corresponding FPP stress mid-level MR image. Corresponding bullseye plots of MBF at (d) rest and at (e) stress, and (f) MPRI calculated using our model.

Observation of the kinetics of tissue enhancement after the injection of a bolus of tracer has been used for the analysis of perfusion and related variables. In general, a gradient of concentration in the exchanging vascular compartment between the arterial and venous ends is represented in models via focus on maintaining the detailed balance between the advective and diffusive exchange processes. Conventionally, this is by considering the exchange in an Eulerian framework, based on considering the exchange within each compartment as a separate unit (e.g., tissue homogeneity (TH) model [1]). Herein, we present a Lagrangian approach to the exchange modeling, such that the blood flowing between compartments is considered as the primary unit, and, thereby, allowing for coarser discretization and more efficient calculations (Figure 1a).

## Methods

Eight patients (age 63 ± 12 years) underwent first-pass perfusion (FPP) rest and regadenoson stress cardiac MRI (CMR) (3T scanner, Tim Trio, Siemens), followed by invasive coronary angiography. Images were obtained at 4 slice locations (the aortic root for the arterial input function (AIF) and 3 short-axis slices of the left ventricle for the wall) using a TurboFLASH readout with centric k-space reordering [2]. A proton density-weighted image was acquired for normalization [3]. Myocardial blood flow (MBF) (mL/g/min) and perfusion reserve index (MPRI) were calculated in endocardial and epicardial areas (total 32 segments) using our method by an expert in the field of MRI blinded to coronary angiography results.

## Results

The results of a representative patient (66 year old man) with history of hypertension, hyperlipidemia, Diabetes Mellitus and known coronary artery disease with prior stents on maximal medical therapy are shown in Figure 1b-f. Coronary angiography was performed via the right femoral artery and demonstrated severe triple-vessel disease with left to right collaterals (Figure 1b). First-pass CMR perfusion imaging demonstrates a delay and decreased delivery to the inferior segments, consistent with ischemia or scar (Figure 1c). There is also suggestion of milder delayed delivery to the anterior segments at stress (Figure 1c). MBFs at rest (at stress) were 0.44 ± 0.08 (0.5 ± 0.08) in the basal and mid anterior segments, 0.35 ± 0.08 (0.17 ± 0.06) in the basal and mid inferior segments, and 0.44 ± 0.03 (0.7 ± 0.12) in the basal and mid anterolateral segments (Figure 1d-e). MPRIs were 1.15 ± 0.12 in the basal and mid anterior segments, 0.52 ± 0.27 in the basal and mid inferior segments, and 1.6 ± 0.28 in the basal and mid anterolateral segments (Figure 1f).

## Conclusions

A novel method combining aspects of TH model and a Lagrangian approach allows quantification of the transit of tracer through myocardial tissue, and may serve to identify physiologically significant coronary artery disease. Larger studies to validate this approach are warranted.

## Funding

NIH R01 HL083309.
